# Supplementary material for: Associations between gut microbiota and personality traits: insights from a captive common marmoset (Callithrix jacchus) colony
Source: Microbiol Spectr. 2025 Nov 18;14(1):e00443-25. doi: 10.1128/spectrum.00443-25 (PMC12772247; doi:10.1128/spectrum.00443-25)
Supplement: Supplemental figures — Figures S1 to S5. [file spectrum.00443-25-s0001.docx]

**Associations between gut microbiota and personality traits: insights from a captive common marmoset (*Callithrix jacchus*) colony**

Huimin Ye^a,b,c^#, Vedrana Šlipogor^d,e,f^#, Buck T. Hanson^a,g^, Joana Séneca ^a,h,i^, Bela Hausmann^h,i^, Craig W. Herbold^a,j^, Petra Pjevac^a,h^ , Thomas Bugnyar^f^, Alexander Loy^a,h^

^a^Division of Microbial Ecology, Centre for Microbiology and Environmental Systems Science,

University of Vienna, Vienna, Austria

^b^Doctoral School in Microbiology and Environmental Science, Centre for Microbiology and

Environmental Systems Science, University of Vienna, Vienna, Austria

^c^APC Microbiome Ireland, University College Cork, Cork, Ireland

^d^Department of Ecology and Evolution, University of Lausanne, Lausanne, Switzerland

^e^The Sense Innovation and Research Center, Lausanne & Sion, Lausanne, Switzerland

^f^Department of Behavioural and Cognitive Biology, University of Vienna, Vienna, Austria

^g^Bioscience Division, Los Alamos National Laboratory, Los Alamos, NM, United States

^h^Joint Microbiome Facility of the Medical University of Vienna and the University of Vienna,

Vienna, Austria

^i^Division of Clinical Microbiology, Department of Laboratory Medicine, Medical University of

Vienna, Vienna, Austria

^j^Te Kura Pūtaiao Koiora, School of Biological Sciences, Te Whare Wānanga o Waitaha,

University of Canterbury, Christchurch, New Zealand

#**Correspondence:** [yehuiminyhm@gmail.com](mailto:yehuiminyhm@gmail.com) (H.Y.), [vedrana.slipogor@](mailto:vedrana.slipogor@gmail.com)unil.ch (V.Š.)

**Supplementary Information**

**a.
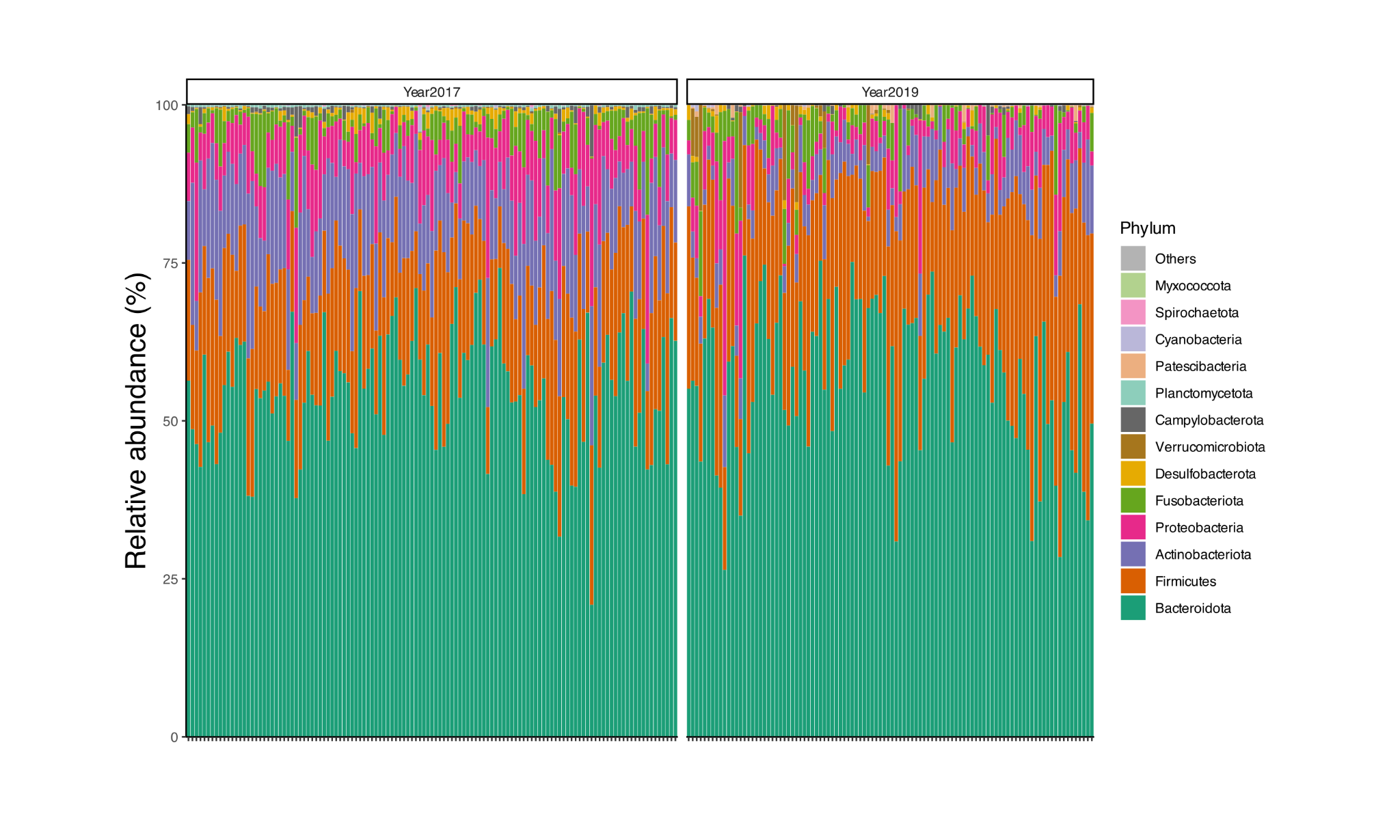
**

**b.
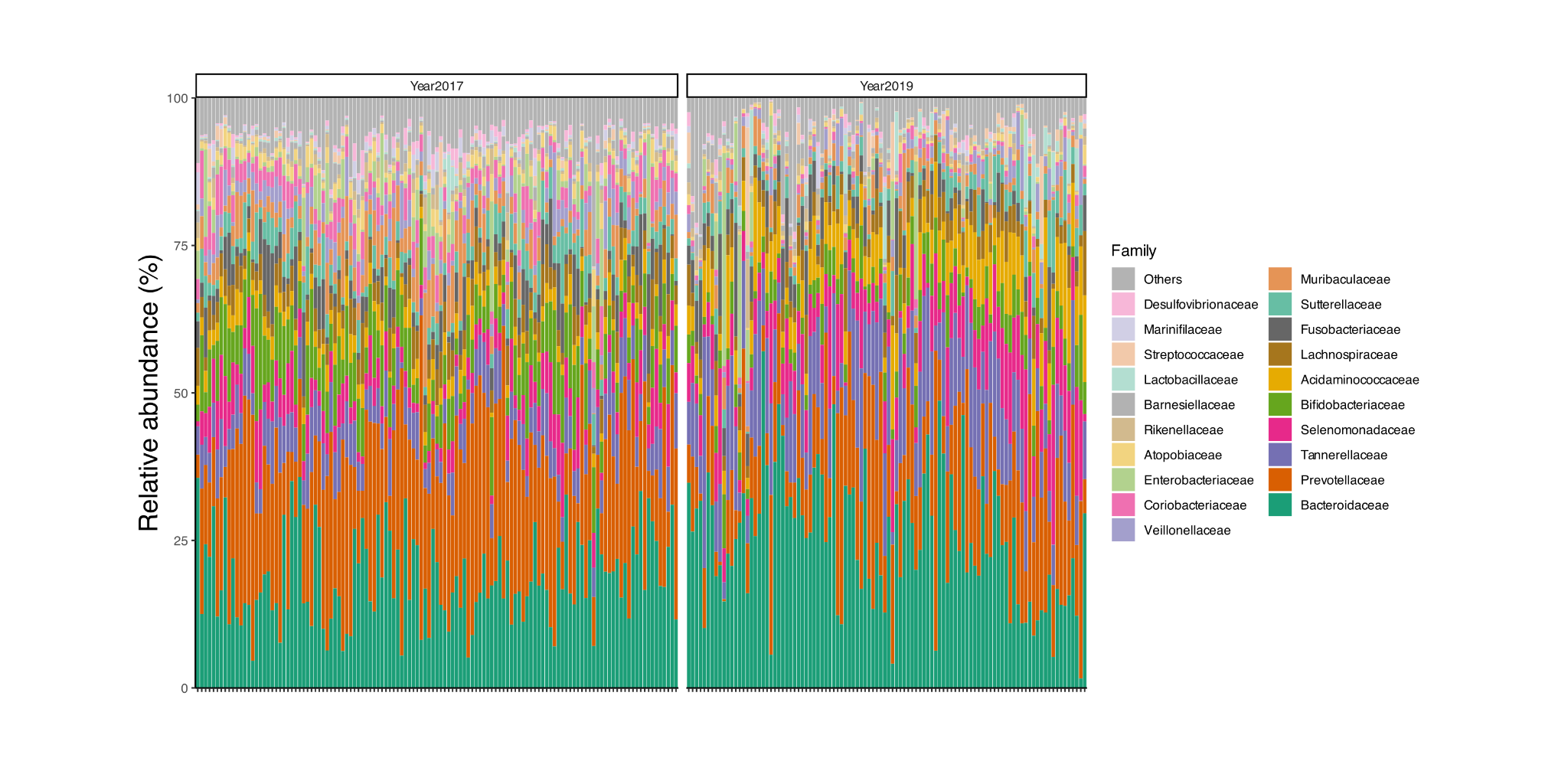
**

**c.
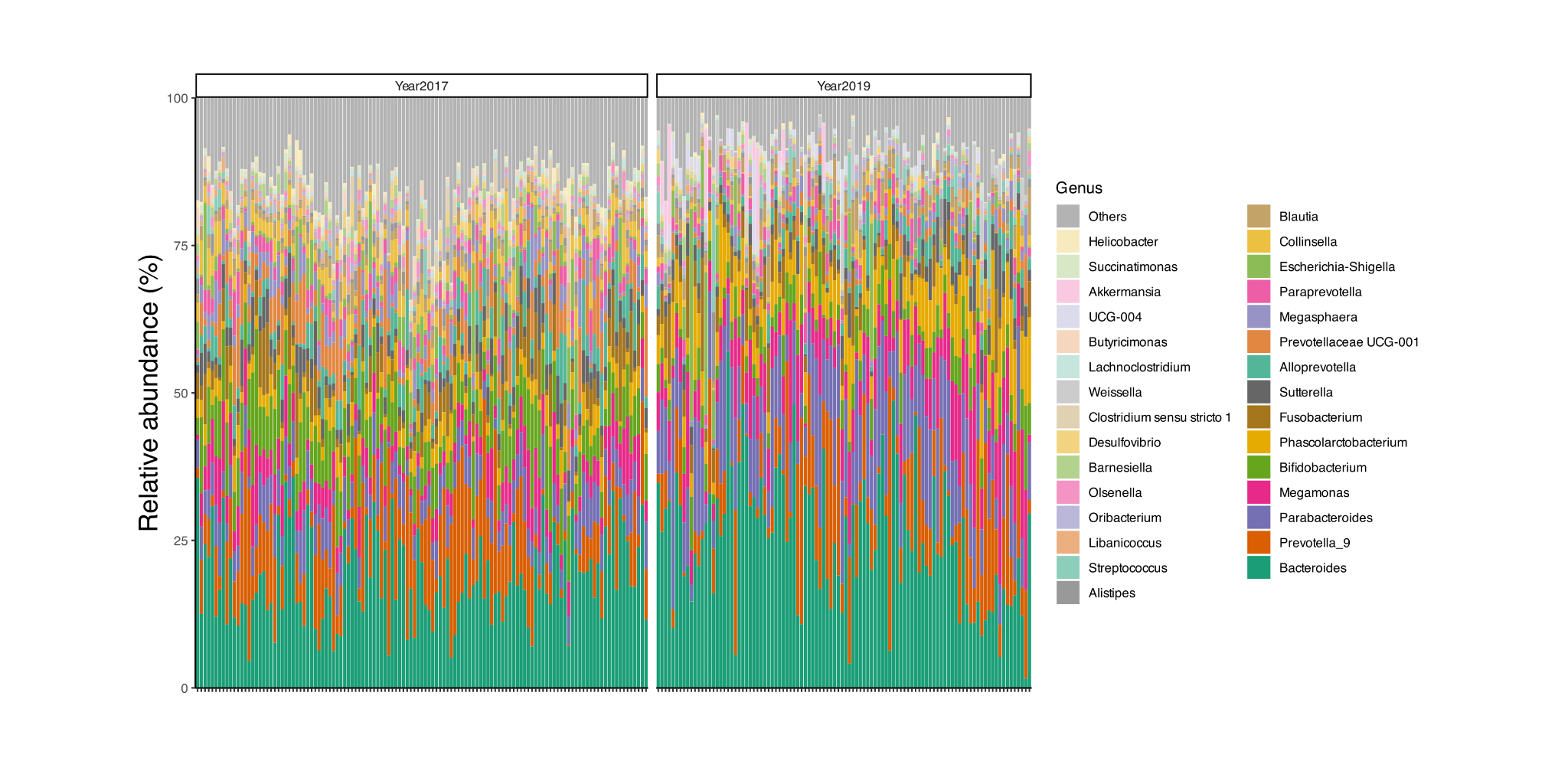
**

**Supplementary Figure S1.** Microbial composition of common marmosets in 2017 (n=123) and 2019 (n=102) at phylum- (**a**), family- (**b**), and genus- (**c**) levels. Each column represents one sample.


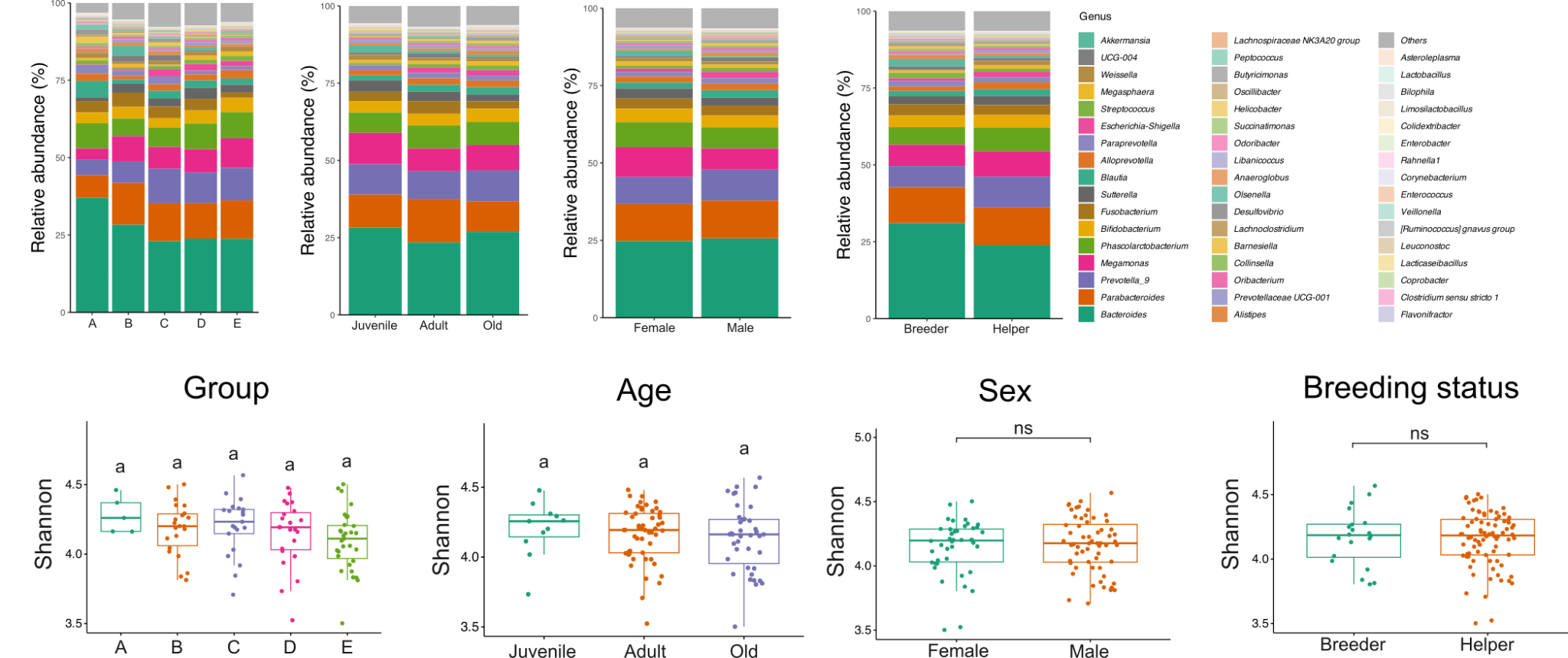


**Supplementary Figure S2. Microbial composition and alpha diversity in different family, age class, sex, and breeding status groups in 2019.** Averaged relative abundances at the genus level showed significant differences associated with family group, age class, sex, and breeding status (upper panel). No significant differences were observed in alpha diversity between different family, age classes, sex, and breeding status groups (lower panel). Shannon’s diversity was compared using Kruskal-Wallis 1-way ANOVA with Dunn's multiple comparison test or *t-test*.

**
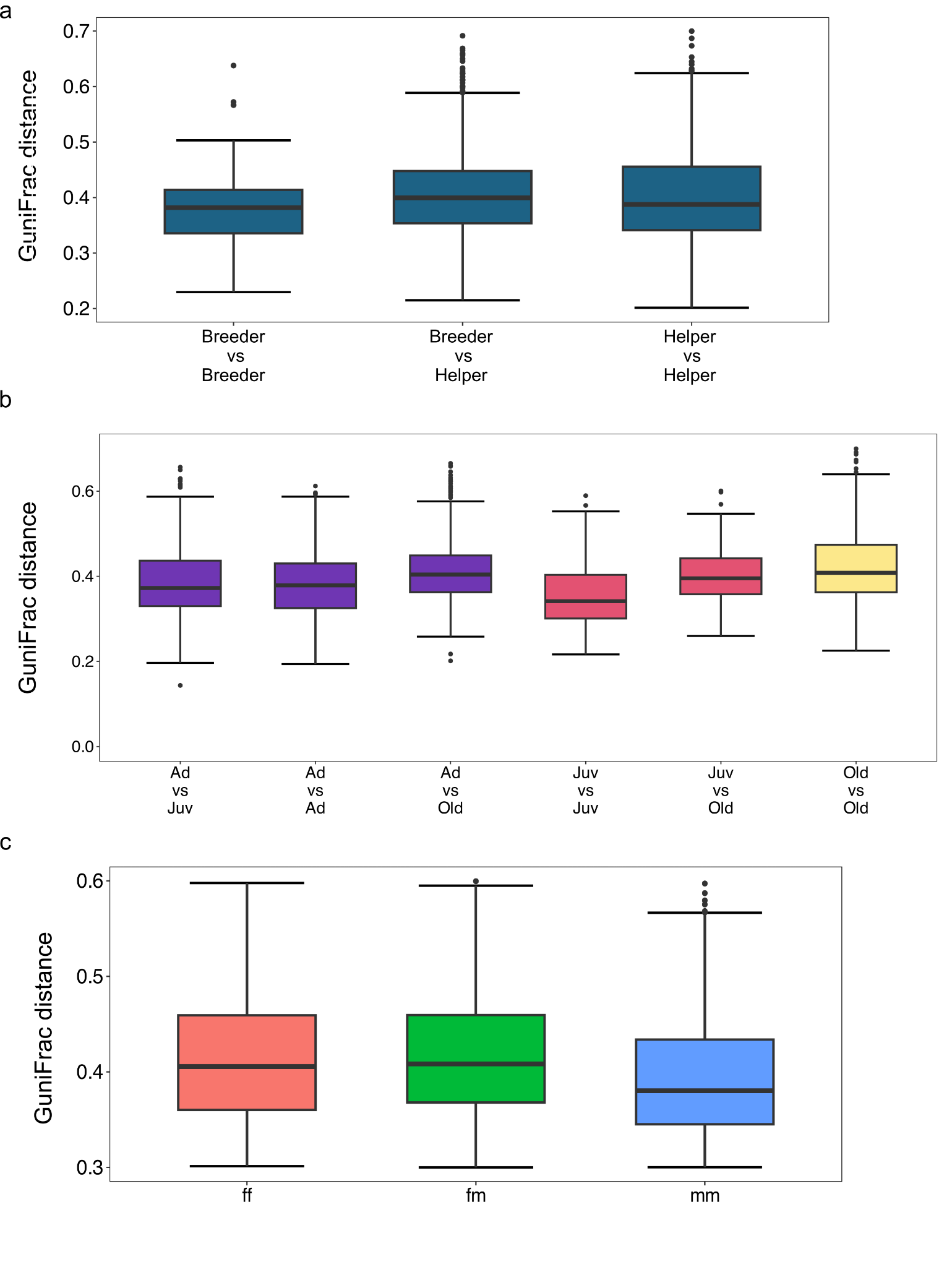
**

**Supplementary Figure S3. Differences in gut microbiota similarity within age class, breeding status, and sex.** GUniFrac distances between group members in different breeding statuses (**a**), age classes (**b**), and sex (**c**). Ad, adult; Juv, juvenile; f, female; m, male.

**
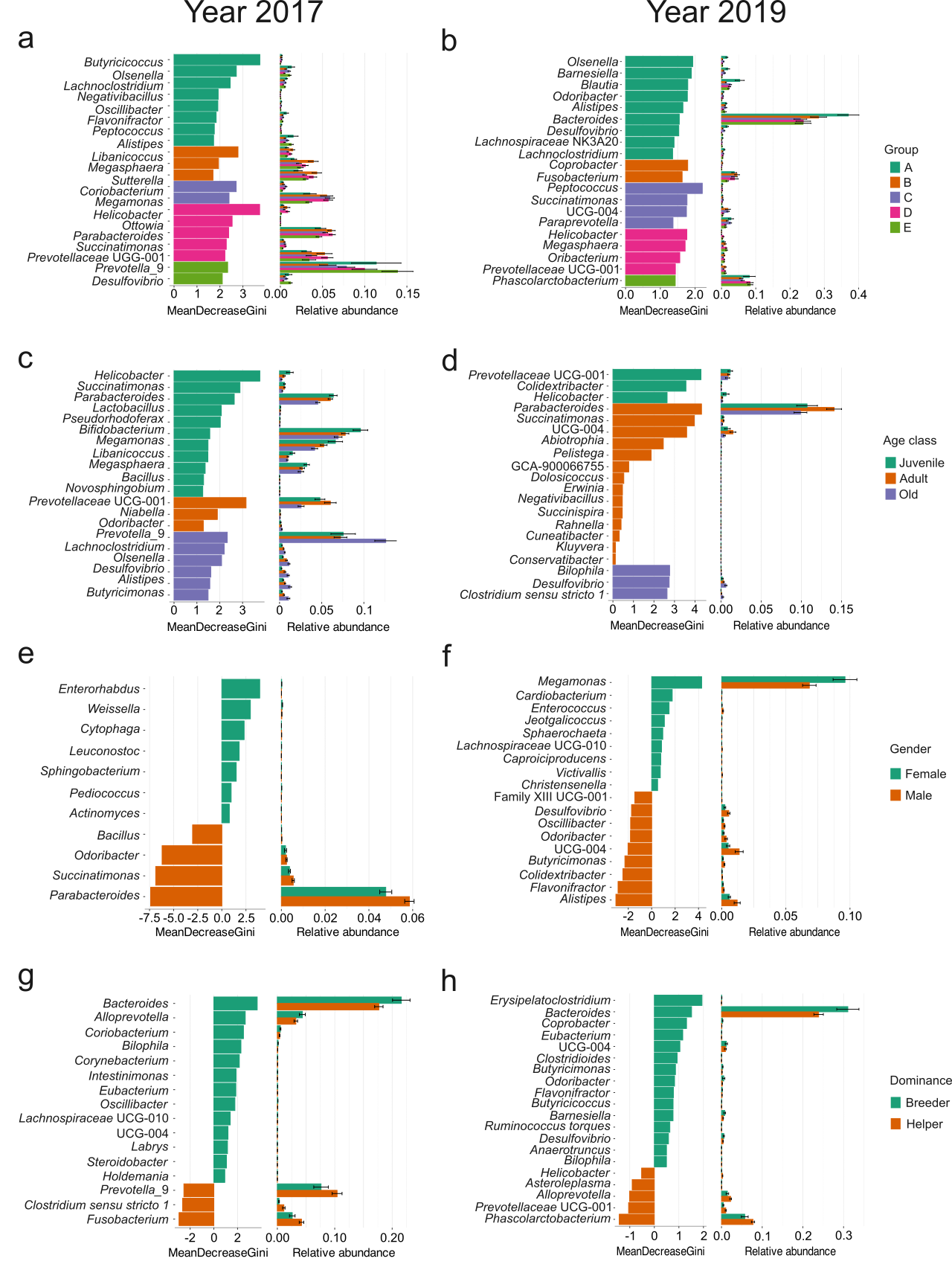
**

**Supplementary Figure S4. Differential genera between social group, age class, sex, and breeding status groups in 2017 and 2019.** Differential taxa were identified at the genus level based on the random forest algorithm.

**
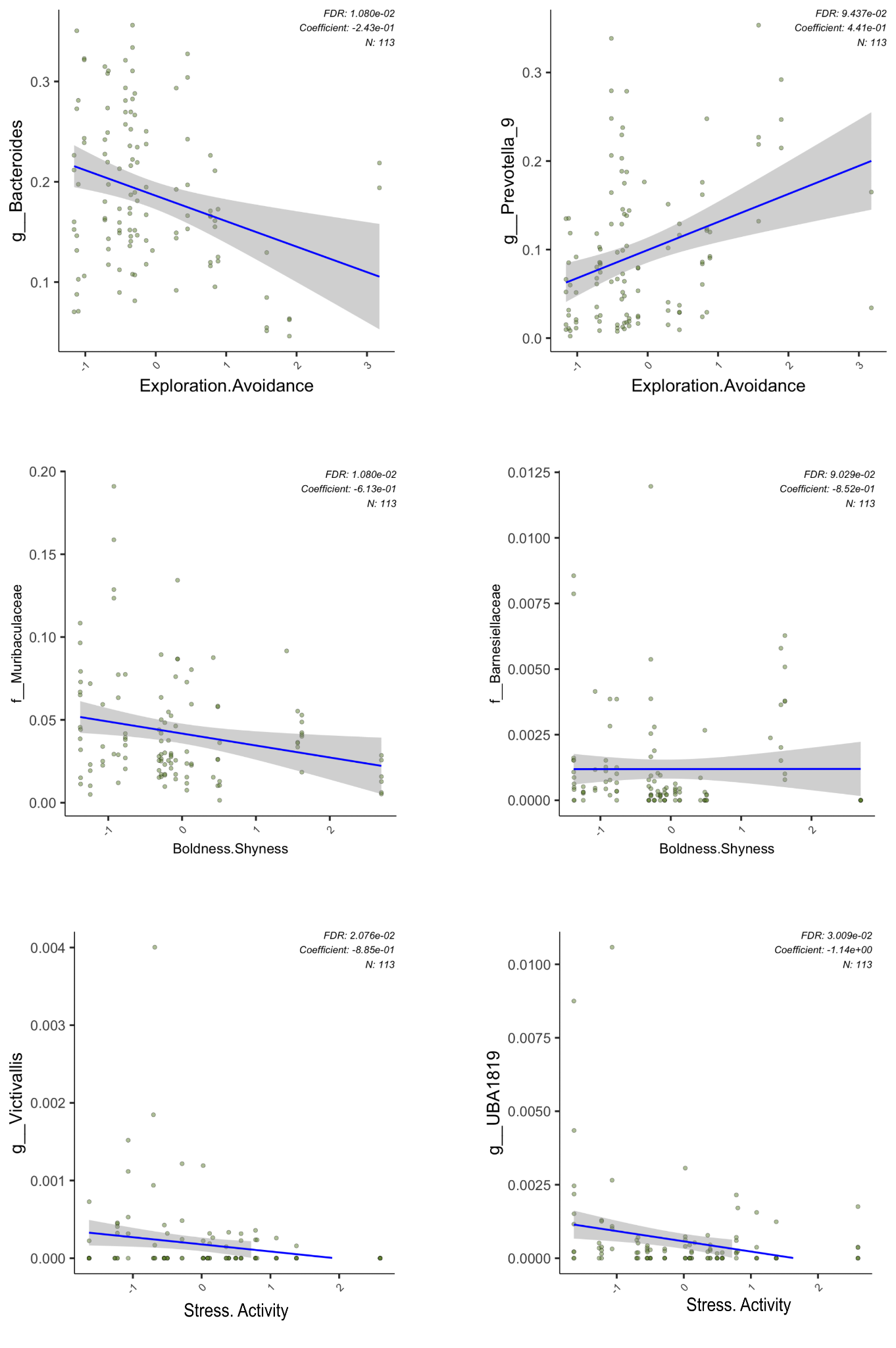
**

**Supplementary Figure S5. Associations between personality scores and gut microbiota composition at the genus level.** Correlations between genus abundances and personality scores for common marmosets. Two taxa with the lowest *p-values* from each personality trait were shown. Family names were displayed when the taxa were unclassified at the genus level. *p* < 0.05 and FDR < 0.05.
